# Metabolic and Pancreatic Effects of Bone Marrow Mesenchymal Stem Cells Transplantation in Mice Fed High-Fat Diet

**DOI:** 10.1371/journal.pone.0124369

**Published:** 2015-04-29

**Authors:** Patricia de Godoy Bueno, Juliana Navarro Ueda Yochite, Graziela Fernanda Derigge-Pisani, Kelen Cristina Ribeiro Malmegrim de Farias, Lucimar Retto da Silva de Avó, Júlio César Voltarelli, Ângela Merice de Oliveira Leal

**Affiliations:** 1 Department of Physiological Science, Center of Biological Sciences and Health, Federal University of São Carlos, São Carlos, São Paulo, Brazil; 2 Department of Biochemistry and Immunology, Ribeirao Preto Medical School, University of São Paulo, Ribeirao Preto, São Paulo, Brazil; 3 Department of Clinical, Toxicological and Bromatological Analyses, Faculty of Pharmaceutical Sciences of Ribeirão Preto, University of São Paulo, Ribeirao Preto, São Paulo, Brazil; 4 Department of Medicine, Federal University of São Carlos, São Carlos, São Paulo, Brazil; Children's Hospital Boston/Harvard Medical School, UNITED STATES

## Abstract

The purpose of this study was to investigate the effects of multiple infusions of allogeneic MSCs on glucose homeostasis and morphometry of pancreatic islets in high- fat diet (HFD) fed mice. *Swiss* mice were fed standard diet (C group) or HFD (HFD group). After 8 weeks, animals of HFD group received sterile phosphate-buffered saline infusions (HFD-PBS) or four infusions of MSCs one week apart (HFD-MSCs). Fasting glycemia (FG) was determined weekly and glucose (GTT) and insulin (ITT) tolerance tests were performed 4, 8, 12, and 16 weeks after the infusions of MSCs. The MSCs transplanted mice were classified as responder (FG < 180 mg/dL, 72.2% of transplanted mice) or non-responder (FG > 180mg/dL, 28.8%) Seven weeks after MSCs infusions, FG decreased in HFD-MSCs responder mice compared with the HFD-PBS group. Sixteen weeks post MSCs infusions, GTT and ITT areas under the curve (AUC) decreased in HFD-MSCs responder mice compared to HFD-PBS group. Serum insulin concentration was higher in HFD-PBS group than in control animals and was not different compared with the other groups. The relative volume of α-cells was significantly smaller in HFD-PBS group than in C group and significantly higher in HFD-MSCs-NR than in HFD-PBS and HFD-MSCs-R groups. Cell apoptosis in the islets was higher in HFD-PBS group than in C group, and lower in HFD-MSCs responder mice than in HFD-PBS group and non-responder animals. The results demonstrate the ability of multiple infusions of MSCs to promote prolonged decrease in hyperglycemia and apoptosis in pancreatic islets and increase in insulin sensitivity in HFD fed mice.

## Introduction

Type 2 Diabetes Mellitus (T2D), the most common form of diabetes (approximately 90% of cases) is caused basically by two pathogenic mechanisms-insulin resistance and secretory dysfunction/decrease of pancreatic β-cells and currently there are experimental, clinical and epidemiological evidences of the involvement of immune and inflammatory mediators in these two mechanisms [1]. Insulin resistance is closely related with obesity. The progression of obesity to insulin resistance and to T2D involves the adaptive expansion of β-cells and increase of insulin secretion, and if this compensation is inadequate, glucose intolerance and T2D develop, with subsequent decline of pancreatic β-cell mass [2,3].

The treatment of T2D is complex and requires nutritional counseling, exercise, several oral drugs and, often, multiple daily insulin injections. Still, treatment of T2D can only ameliorate hyperglycemia or temporarily improve the response to insulin in target tissues. In addition, adherence to therapy is usually low and most patients maintain hyperglycemia, which is the major factor responsible for the onset of the chronic and severe complications of diabetes [4]. Therefore, the development of new preventive and therapeutic strategies for T2D is essential. The interest in regenerative therapeutics for T2D was initially motivated by the importance of preserving β-cell mass and function.

The regenerative cellular therapy, in particular with multi/pluripotent cells, has been investigated as a potential therapeutic strategy for T2D [5]. Among them, mesenchymal stem cells (MSCs) due to their immunoregulatory properties are relevant therapeutic candidates [6,7].

Bone marrow (BM) is an important source of easily obtained adult stem cells that include hematopoietic stem cells, mesenchymal stromal stem cells, and endothelial progenitor cells [8]. MSCs are one of the most important multipotent adult stem cells, which can be extensively culture-expanded, are undifferentiated, self-renewable, have low immunogenicity and their clinical utilization involves few ethical concerns [6].

MSCs are able to modify the microenvironment of injured tissues contributing to tissue repair and regeneration through the secretion of cytokines, anti-inflammatory and anti-apoptotic molecules with trophic and immunomodulatory functions [9,10,11,12].

Several studies have shown that MSCs transplantation decreased blood glucose levels and promoted regeneration of pancreatic islet of diabetic animals [13,14,15,16,17,18,19,20,21]. However, these findings were still not adequate to explain the therapeutic contribution of MSC to T2D. Most pre-clinical studies of type 2 diabetes use transgenic manipulations or streptozotocin-induced diabetes as experimental models to evaluate the metabolic effects MSCs infusions [14,22,23]. Nevertheless, these animal models do not reflect the pathogenesis of the human disease which is complex and closely associated with obesity. However, so far, the effects of MSCs infusion in the high fat diet (HFD)-induced diabetes model have been unknown.

Morphometric studies of pancreatic islets have been made since the 50's and since then have helped unravel the complex relationship between the different cell types that compose them as well as their relationship with physiological and pathological conditions, especially diabetes mellitus type 1 and type 2 [24,25].

The purpose of this study was to investigate the effects of multiple infusions of allogeneic BM MSCs on glucose homeostasis and morphometry of pancreatic islets in HFD-induced hyperglycemia in *Swiss* mice.

## Materials and Methods

### Animals and experimental groups

**Fig 1:**
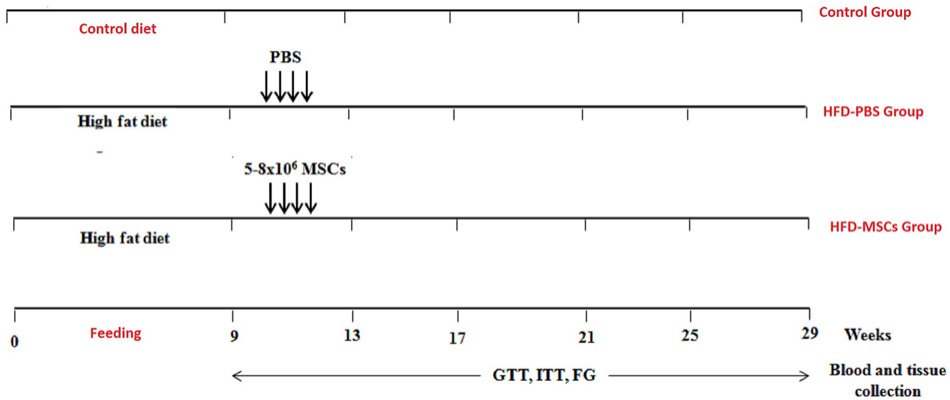
Experimental groups and study design. Eight weeks after high fat-diet or standard diet regimen (control group), mice received 4 weekly intraperitoneal (i.p) infusions of PBS (HFD-PBS group) or 5–8 x 106 BM-MSC (HFD-MSC group). Fasting glycemia (FG) was determined every week and glucose (GTT) and insulin (ITT) tolerance tests were performed in the 9^th^, 17^th^, 21^st^, 25^th^, and 29^th^ week of the experimental period.

Four week-old male *Swiss* mice (State University of Campinas Central Breeding Center, Campinas, São Paulo, Brazil), were acclimated in individual cages under controlled temperature, humidity and lighting (12-h dark/light cycle) and with free access to water and standard rodent chow. After 7 days, the animals were randomly assigned into 2 groups, Control group (C) fed standard rodent chow and High-fat diet group (HFD) fed 60% of Kcal as fat (PragSoluções, São Paulo, Brazil). After 8 weeks, animals of HFD group were randomly divided into 2 groups: HF mice, which received sterile phosphate-buffered saline (PBS) infusions (HFD-PBS) and HF mice, which received multiple MSCs infusions (HFD-MSCs), (Fig 1).

Animal protocols were approved by the Ethic Committee of the Federal University of São Carlos (Approval ID number 053/2008).

### Fasting glycemia, serum insulin determinations and glucose (GTT) and insulin (ITT) tolerance tests

Fasting glycemia (FG) was determined after 8 weeks of the diet regimen. The animals from which FG was ≥ 180 mg/dL were considered hyperglycemic [26]. Fasting glycemia was also determined weekly after MSCs infusions.

GTT and ITT were performed after 8 weeks of the diet regimen and 4, 8, 12, and 16 weeks after the MSCs infusions. After overnight fasting, unanesthetized mice were injected with 1.5g of 50% glucose solution per kg of body weight (BW) by intraperitoneal (i.p) route. Blood samples were collected before glucose injection and 30, 60, 90, and 120 min after the injection. ITT was performed after overnight fasting, in unanesthetized mice injected i.p. with human insulin 0.75 U/kg BW. Blood samples were collected before injection and 15, 30, 60, and 90 min after insulin challenge.

Blood glucose samples were collected from the tail vein. Blood glucose levels were measured by Accu-Check glucose meter (Roche Diagnostic, Indianapolis, USA) and serum insulin concentration was determined by ELISA (Millipore Corporation, Billerica, MA, U.S.A.) according to the manufacturer’s protocol.

### Isolation, culture and characterization of bone marrow MSCs

The bone marrow cells were isolated from the tibias and femurs of male *Wistar* rats, aged 6–8 weeks. After washing and centrifugation, cells were resuspended in a α-minimum medium (Gibco, Auckland, New Zealand) supplemented with 15% of fetal bovine serum (Hyclone, Logan, UT, USA) and 100 μg/ml penicillin (Gibco), 100 μg/ml streptomycin (Gibco) and 2 mM L‐glutamine (Gibco), and plated in a 150x20mm dish (density of 5 x 10^6^ nucleated cells/dish). The cells were incubated in a humidified atmosphere containing 95% air and 5% CO_2_ at 37^°^C. The non-adherent cells were removed by changing the medium after 3-day culture. Confluent primary cultures were washed with PBS and lifted by incubation with trypsin (Sigma‐Aldrich, Saint Louis, MO, USA) at 37°C for 5 minutes. RPMI1640 (Gibco), supplemented with 10% fetal bovine serum (HyClone, Logan, UT, Canada) was added to neutralize the excess trypsin. The cells were centrifuged and seeded into a 150x20mm dish (density of 5x10^6^ nucleated cells/dish). Subsequent passages were performed similarly until the fifth passage.

To characterize MSCs, one aliquot of trypsinised MSCs from the fifth passage was stained with phycoerythrin (PE)-conjugated monoclonal antibodies against CD31, CD45 or fluorescein isothiocyanate (FITC)-conjugated monoclonal antibodies against CD 11b, CD44, and CD29 (Becton-Dickinson/BD, San Jose, CA, USA) for 15 minutes at room temperature. Stained cells were washed and analyzed immediately on a FACSort flow cytometer using CellQuest software (BD).

Adherent cells were further characterized according to their *in vitro* osteogenic and adipogenic differential potential. Osteogenic differentiation was induced by culturing confluent MSCs for 3 weeks in α-minimum medium (Gibco) supplemented with 7.5% fetal bovine serum (Hyclone, Logan, UT, USA), 1μM dexamethasone, 200 μM ascorbic acid (Sigma‐Aldrich), 10mM β-glycerophosphate (Sigma‐Aldrich). To observe calcium deposition, cultures were analyzed by von Kossa staining [27]. Adipogenic differentiation was induced by culturing confluent MSCs for 2 weeks in α-minimum medium (Gibco) supplemented with 15% fetal bovine serum (Hyclone, Logan, UT, USA), 100mM dexamethasone (Prodome, Campinas, SP, Brazil) 10μg/mL insulin (Sigma‐Aldrich), and 100μM indomethacin (Sigma‐Aldrich). The cells were then analyzed by Sudan II-Scarlat staining [28].

### Multiple administration of MSCs

Bone marrow MSCs between fourth to fifth passages were used for multiple infusions in HFD mice. HFD-MSCs group received four i.p. infusions of 5 – 8x10^6^MSCs resuspended in 200μL of PBS, with one week of interval. HFD-PBS group received 200μL of PBS i.p. (Fig 1).

### Pancreas immunohistochemistry

Sixteen weeks after the last MSCs infusion, animals were euthanized and pancreas was rapidly removed, fixed in 10% neutral-buffered formalin, dehydrated through graded ethanol passages, cleared in xylene and embedded in paraffin wax. Five μm sections were deparaffinized and rehydrated in a graded series of ethanol washes. Subsequently, 3 discontinuous sections of 5μm pancreas sample were obtained and stained by hematoxylin and eosin (HE) and immunodetection of insulin, glucagon, Ki67 and caspase. Only for Ki67 and caspase detection, antigen retrieval was performed using citrate buffer. Endogenous peroxidase was quenched by hydrogen peroxide 3% (Peroxidase-Blocking reagent, DAKO Cytomation, Fort Collins, CO, USA). Then, sections were incubated with PBS/ BSA 1% to prevent unspecific staining. Next, primary antibodies were applied to the sections. In this study, four primary antibodies diluted in PBS/1%BSA were used: 1) rabbit polyclonal anti-insulin (dilution 1:100; Santa Cruz Biotechnology, CA, USA), 2) mouse monoclonal anti-glucagon (dilution 1:2000; Abcam, Cambridge, UK), 3) rabbit polyclonal anti-caspase (dilution 1:500; Abcam, Cambridge, UK) or 4) rabbit monoclonal anti-Ki67 (dilution 1:100; Abcam, Cambridge, UK). Sections were then incubated with LSAB^TM^+ Kit/HRP (DAKO Cytomation, Fort Collins, CO, USA). The slides were stained with 3.3’diaminobenzidine (DAB—DAKO) according to the manufacturer’s instructions. Finally the sections were and counterstained with Harrys hematoxylin stain. Images were captured and analyzed by a system composed of a video cam (Carl Zeiss AxionCam-MRc) coupled to an Axion Vision Vert A1 microscope (Car Zeiss GmbH, Jena, German), linked to a microcomputer with a Axiovision 4.8 software. Captures were performed using X20 objective magnification.

### Determination of islet size and quantification of β and α cells volume

For morphometric pancreas analysis, fifteen islets from each mouse were randomly chosen. Each islet was evaluated to obtain the total islet area and the area positive for insulin or glucagon within each islet using Image-Pro Plus software (Media Cybernetics, Silver Spring, MD). Then, the relative volume of β-cell or α-cell was calculated as the percentage area positive for insulin or glucagon within the islets [25].

### Proliferation and apoptosis

Pancreatic islet apoptosis and proliferation was expressed as caspase-3 or Ki67 positive cells, respectively, per islet area. Software Image-Pro Plus (Media Cybernetics, Silver Spring, MD) was used to measure areas and Image J (National Institutes of Health, Bethesda, MD, USA) was used to count the positive cells.

### Statistical analysis

Data are presented as mean ± SEM. Statistical analysis was done by unpaired and paired Student’s *t* tests and analysis of variance (ANOVA). Tukey’s multiple comparisons test was used for post hoc analysis of between-group comparisons. Data are presented as means ± SEM. P values < 0.05 were considered statistically significant.

All statistical analyses were performed by GraphPad Prism 5.0 software (GraphPad software, Inc., California, USA)

## Results

### Characterization of allogeneic bone marrow derived MSCs

**Fig 2:**
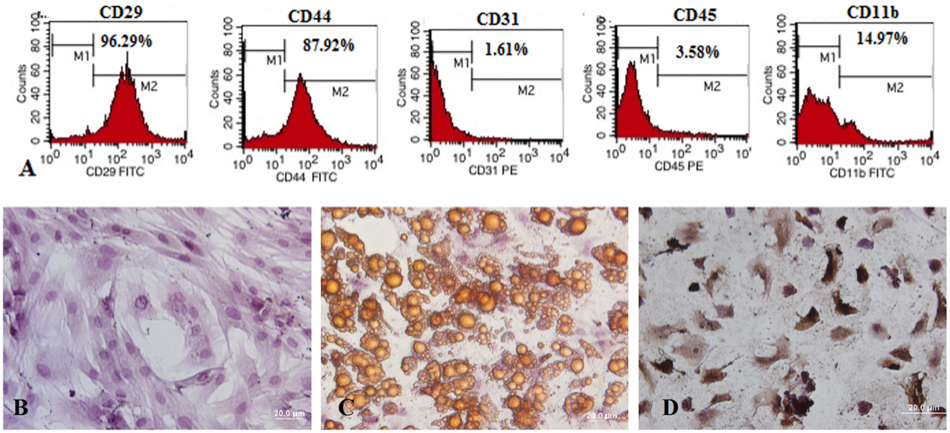
Bone marrow-derived MSCs isolated from *Wistar* rats. Immunologic phenotypes of MSCs (A). Passage five plastic adherent cells cultured in alpha-MEM supplemented with 15% fetal bovine serum (B) and differentiated into adipogenic (C) or osteogenic (D) lineages.

Allogeneic BM-MSCs expressed typical mesenchymal cell markers (Fig 2A) as previously described [29]. MSCs stained positive for CD44 and CD29 while negative for CD31, CD45 and CD11b. MSCs were further characterized by their osteogenic and adipogenic differential potential (Fig 2B–2D).

### Metabolic characteristic of the animals

**Fig 3:**
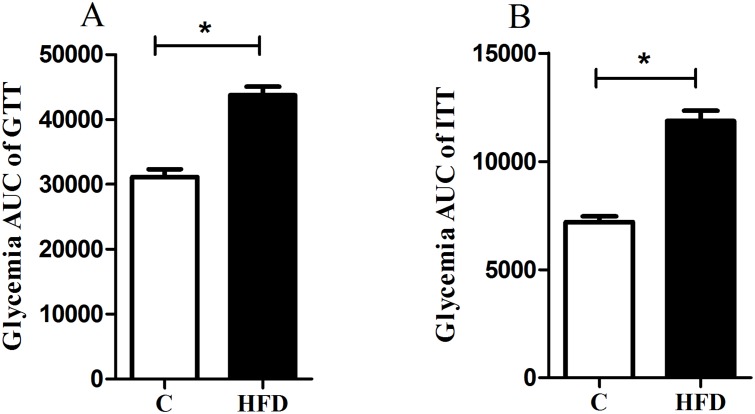
Glucose Tolerance Test (GTT) (A) and Insulin Tolerance Test (ITT) glycemia responses (B) increase in high fat diet-fed mice. Glycemia AUC, area under the curve (AUC) analysis of glycemia profile of GTT and ITT after eight weeks of diet regimen. Values are expressed as mean ± SEM (10–28 mice/group). * p < 0.05, control group (C) *vs*. high fat diet group (HFD).

Eight weeks after the HFD regimen, mice fed high-fat diet had significantly higher FG than that of control animals (210.5±6.8 vs 133.22±4.8 mg/dL, p <0.05) until the end of the experimental period. These animals had significantly higher glycemia response to both glucose and insulin injections than that of control mice (Fig 3A and 3B).

### MSCs multiple injections decrease blood glucose levels and improve glucose and insulin response

**Fig 4:**
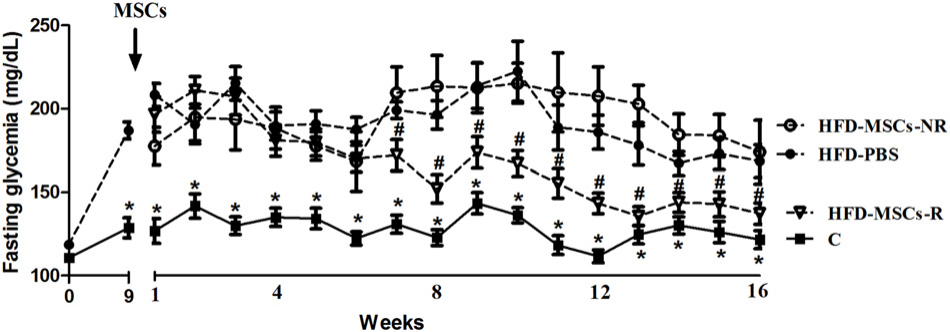
Fasting glycemia decrease since the 7^th^ week post-MSCs infusion. Fasting glycemia of HFD-MSCs-R mice was significantly lower compared to HFD-PBS since the 7^th^ week post- MSC infusion. Values are expressed as mean ± SEM (5–13 mice/group). * p < 0.05, control group (C) *vs*. high fat diet group (HFD-PBS); ^#^ p < 0.05, HFD-MSCs-R *vs*. HFD-PBS.

**Fig 5:**
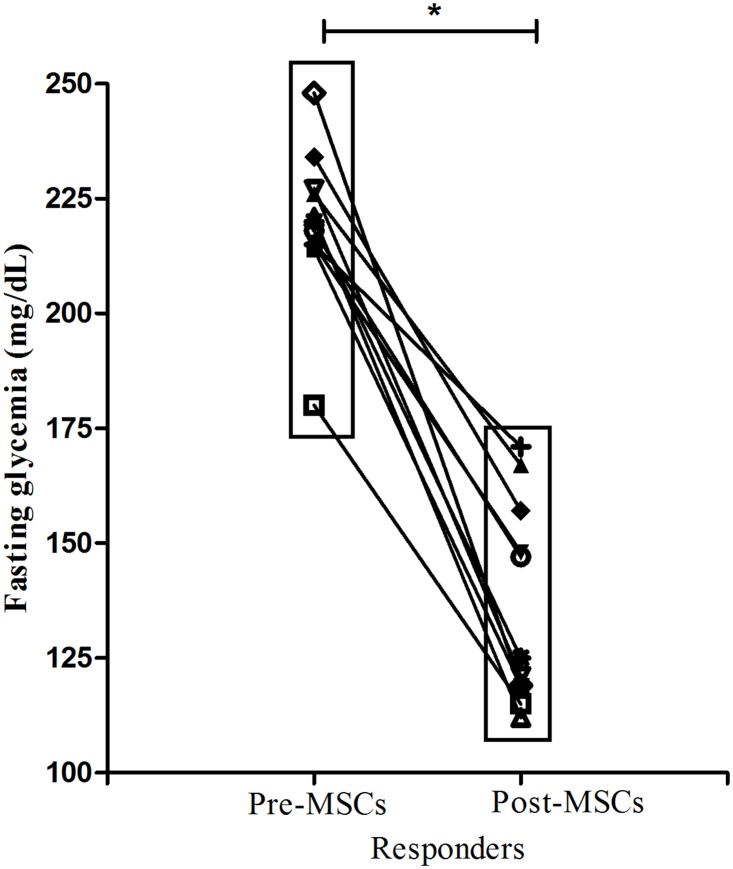
Fasting glycemia decrease post-MSCs infusion. Sixteen weeks after the 4^th^ infusion of BM-MSCs, 72.2% (n = 13) of the animals attained fasting glycemia lower than 180 mg/dL (responders). Post- infusion fasting glycemia of these mice was significantly lower than their pre-infusion values. * p < 0.05, pre-*vs*. post-infusion of BM-MSCs fasting glycemia.

Sixteen weeks after BM-MSCs fourth infusion, 72.2% (n = 13) of the animals exhibited fasting glycemia lower than 180 mg/dL (henceforth called responders or HFD-MSCs-R), while 27.8% (n = 5) did not respond to MSCs therapy—non-responders (or HFD-MSCs-NR). Fig 4 shows the evolution of fasting glycemia in each group throughout the entire experimental period. Fasting glycemia of HFD-MSCs-R mice was significantly lower compared HFD-PBS since the fourth week post- MSCs infusion. Post-treatment fasting glycemia of HFD-MSCs-R was significantly lower than their pre-treatment values (140.5 ± 22.4 *vs* 214.0 ± 20.8, p< 0.05, Fig 5).

Body weight of HFD fed animals was significantly higher than control animals beginning at the 8^th^ week until the end of the experimental period. At the end, the body weight of HFD-PBS, HFD-MSCs-R and HFD-MSCs-NR was not different (Table 1).

**Table 1:** Body Weight (g).

Groups	Initial	8^th^ week (pre-MSCs infusions)	Final
C	24.4 ± 0.4	41.7 ± 1.2	55.8 ± 2.2
HDF-PBS	26.0 ± 0.4	47.7 ± 1.5*	68.0 ± 2.2*
HFD-MSCs-R			71.3 ± 2.8
HFD-MSCs-NR			65.4 ± 2.0

Values are expressed as mean ± SEM (n = 5–13 mice/group).

*p < 0.05, HFD-PBS *vs*. C.

**Fig 6:**
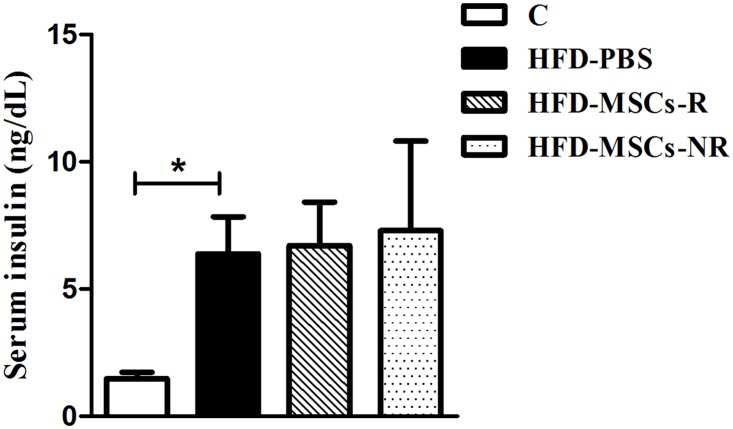
Serum insulin levels increase in high-fat-diet fed mice. Values are expressed as mean ± SEM (5–13 mice/group). * p < 0.05, control group (C) *vs*. high fat diet infused with PBS (HFD-PBS).

Serum insulin levels were significantly higher in HFD-PBS than in C (Fig 6). However, there was no difference among HFD-PBS, HFD-MSCs-R and HFD-MSCs-NR groups.

**Fig 7:**
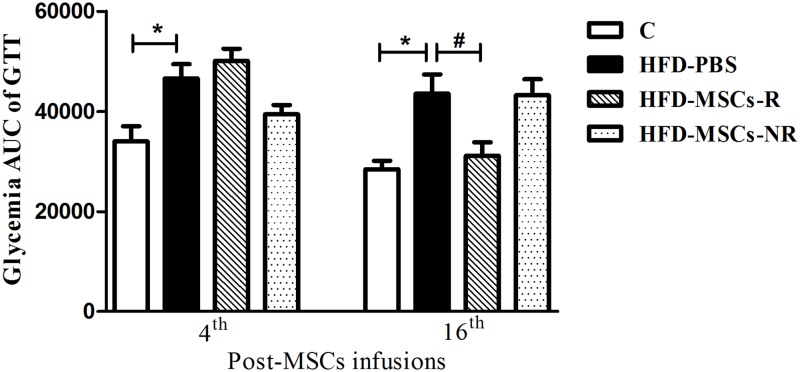
Glucose Tolerance Test (GTT) glycemia response decrease post-MSCs infusion. Only in the 16^th^ week post-MSC infusions, the glycemia response to glucose injection was significantly lower in HFD-MSCs-R when compared to HFD-PBS group. Values are expressed as mean ± SEM (5–13 mice/group). * p < 0.05, control group (C) *vs*. HFD-PBS; ^#^ p < 0.05, HFD-MSCs-R *vs*. HFD-PBS.

Glycemia response to glucose injection (GTT) was significantly higher in HFD-PBS than in C in the fourth week post-MSCs or PBS infusions. However, there was no difference among HFD-PBS, HFD-MSCs-R and HFD-MSCs-NR groups. The same pattern was observed in the 8^th^ and 12^th^ weeks post-MSCs or PBS infusions (data not shown). In the 16^th^ week post-MSCs or PBS infusions, the glycemia response to glucose injection was significantly lower in HFD-MSCs-R than in HFD-PBS group (Fig 7).

**Fig 8:**
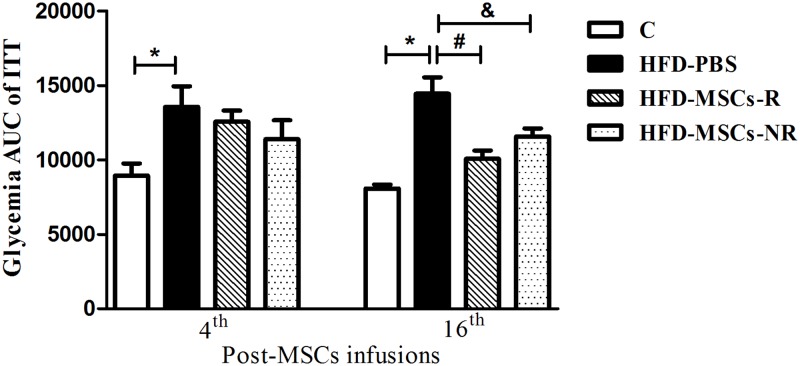
Insulin Tolerance Test (ITT) glycemia response decrease post-MSCs infusion. Only in the 16^th^ week post-MSC infusions, the glycemia response to insulin injection was significantly lower in both HFD-MSCs-R and HFD-MSCs-NR compared to HFD-PBS group. Values are expressed as mean ± SEM (5–13 mice/group). * p < 0.05, control group (C) *vs*. HFD-PBS; ^#^ p < 0.05, HFD-MSCs-R *vs*. HFD-PBS; ^&^ p < 0.05, HFD-MSCs-NR *vs*. HFD-PBS.

Glycemia response to insulin injection (ITT) was significantly higher in HFD-PBS than in C at the fourth week post-MSCs or PBS infusions. Nevertheless, there was no difference among HFD-PBS, HFD-MSCs-R and HFD-MSCs-NR groups. The same pattern was observed in the 8^th^ and 12^th^ weeks post-MSCs or PBS infusions (data not shown). In the 16^th^ week post-MSCs or PBS infusions, the glycemia response to insulin was significantly lower in both HFD-MSCs-R and HFD-MSCs-NR than in HFD-PBS group (Fig 8).

### Pancreatic islet analysis

**Fig 9:**
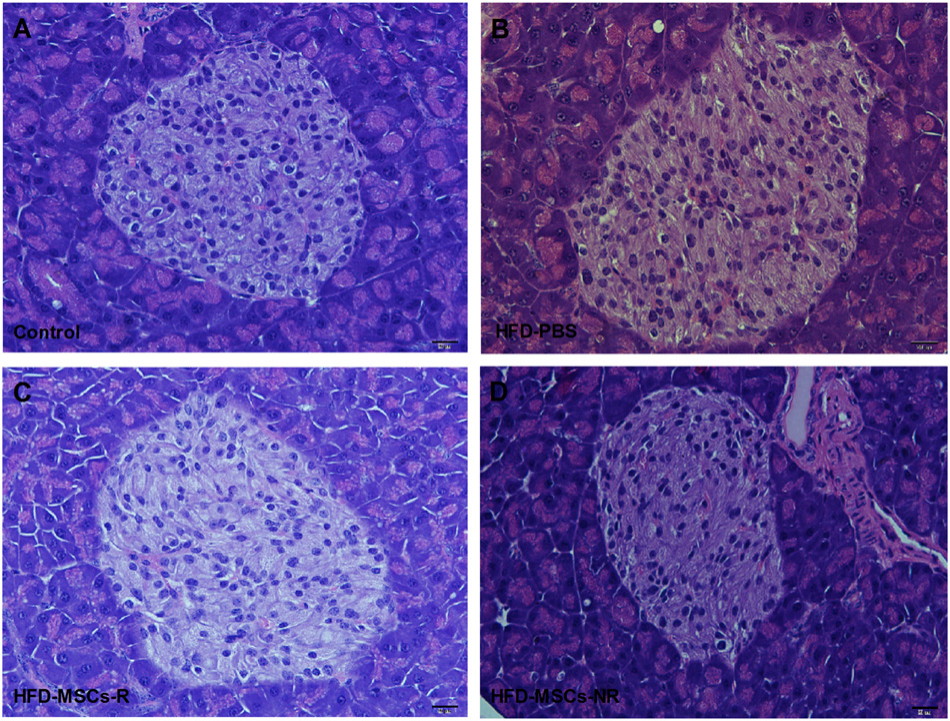
Morphological features of pancreatic islet by hematoxylin and eosin staining. Representative images of pancreas section stained for hematoxylin and eosin of control group (A) and HFD-PBS (B), HFD-MSCs-R (C), and HFD-MSCs-NR (D) groups.

Pancreatic islets showed no morphological changes, maintaining their typical round or oval shapes. No inflammatory cells (insulitis) were observed in the pancreatic islets (Fig 9)

**Fig 10:**
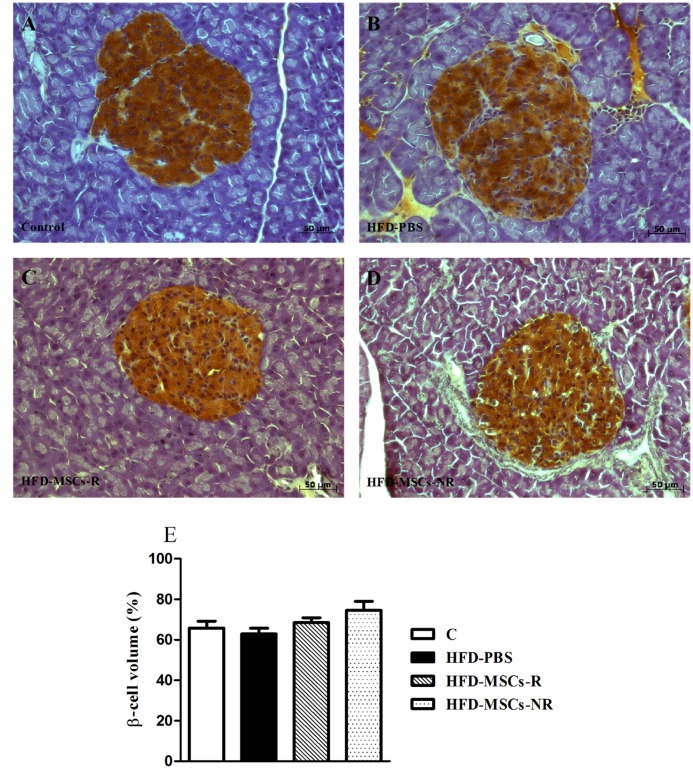
Islet areas and relative volumes of β-cell did not differ among the groups. Representative images of pancreas section stained for insulin of control group (A) and HFD-PBS (B), HFD-MSCs-R (C), and HFD-MSCs-NR (D) groups. Quantitative data correspond to mean ± SEM (5–13 mice/group) (E).

**Fig 11:**
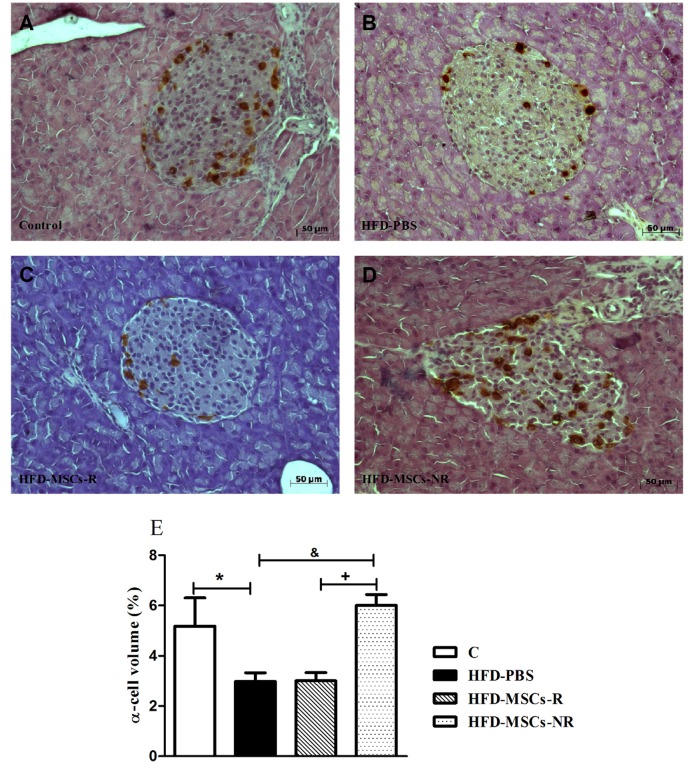
Relative volumes of islet α-cell decrease in HFD-PBS and increase in HFD-MSCs-NR. Representative images of pancreas section stained for glucagon of control group (A) and HFD-PBS (B), HFD-MSCs-R (C), and HFD-MSCs-NR (D) groups. Quantitative data correspond to mean ± SEM (5–13 mice/group) (E). * p < 0.05, control group (C) *vs*. HFD-PBS; ^+^ p < 0.05, HFD-MSCs-NR *vs*. HFD-PBS; ^&^ p < 0.05, HFD-MSCs-NR *vs*. HFD-MSCs-R.

According to the morphometric analysis of the islets, the total islet areas and the relative volumes of β-cells were not different among the groups (Fig 10A–10E). However, the relative volumes of α-cells were significantly smaller in HFD-PBS group than in C and significantly higher in HFD-MSCs-NR than in HFD-PBS and HFD-MSCs-R groups (Fig 11A–11E).

**Fig 12:**
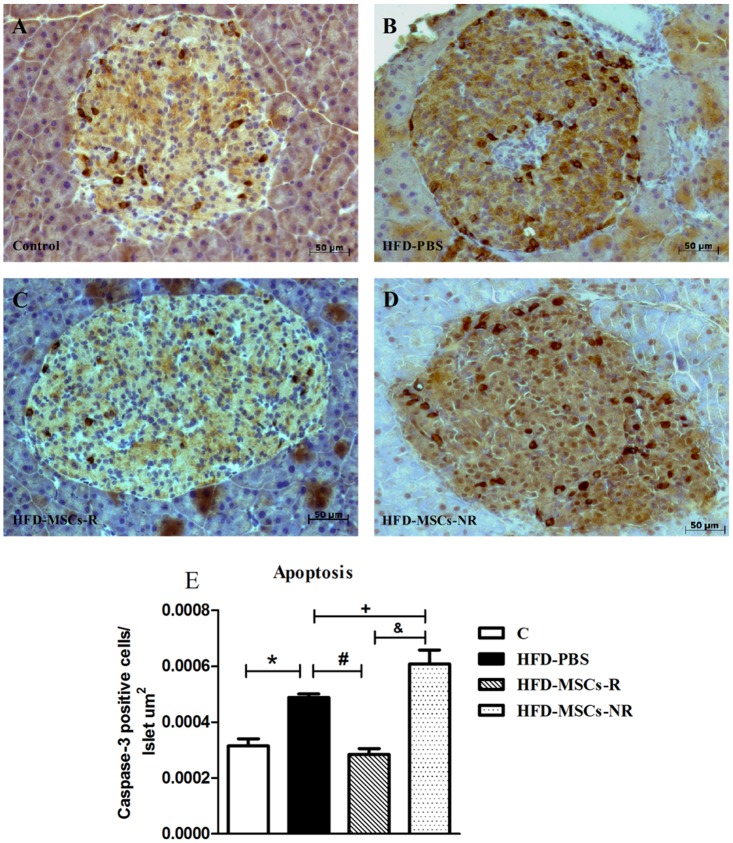
Islet apoptosis increases in HFD-PBS and HFD-MSCs-NR and decreases in HFD-MSCs-R. Representative images of pancreas section stained for caspase-3 of control group (A) and HFD-PBS (B), HFD-MSCs-R (C), and HFD-MSCs-NR (D) groups. Quantitative data correspond to mean ± SEM (5–13 mice/group) (E). * p < 0.05, control group (C) *vs*. HFD-PBS; ^#^ p < 0.05, HFD-MSCs-R *vs*. HFD-PBS; ^+^ p < 0.05, HFD-MSCs-NR *vs*. HFD-PBS; ^&^ p < 0.05, HFD-MSCs-NR *vs*. HFD-MSCs-R.

Apoptosis in islet cells was significantly higher in HFD-PBS group than in C and in HFD-MSCs-NR group compared with HFD-PBS and HFD-MSCs-R groups. However, the number of apoptotic cells was reduced in HFD-MSCs-R group when compared with HFD-PBS group (Fig 12A–12E).

**Fig 13:**
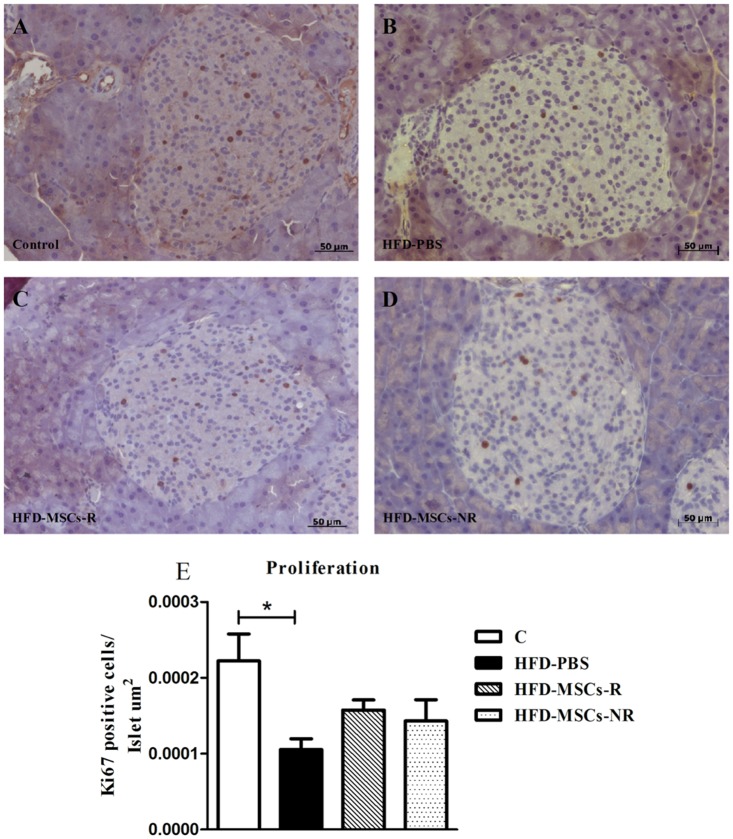
Islet proliferation decreases in high fat diet-fed mice. Representative images of pancreas section stained for Ki67 of control group (A) and HFD-PBS (B), HFD-MSCs-R (C), and HFD-MSCs-NR (D) groups. Quantitative data correspond to mean ± SEM (5–13 mice/group) (E). * p < 0.05, control group (C) *vs*. HFD-PBS.

Regarding islet cellular proliferation, it was significantly smaller in HFD-PBS group than in C. No other difference among the groups was observed (Fig 13A–13E).

**Fig 14:**
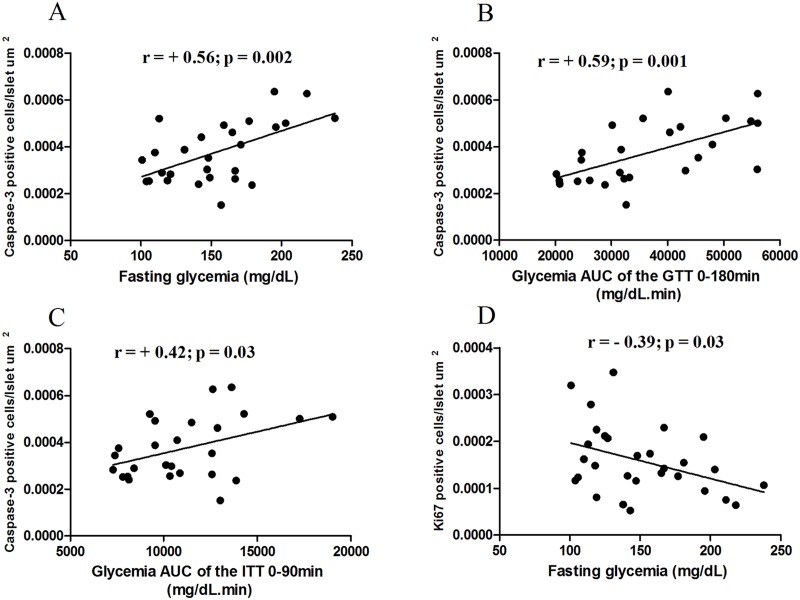
Correlation analysis. Islet apoptosis was positively correlated with fasting glycemia (A) and glucose tolerance test (GTT) (B) and insulin tolerance test (ITT) glycemia responses (C). Islet proliferation was negatively correlated with fasting glycemia (D).

Apoptosis in islets was positively correlated with fasting glycemia (r = 0.56; p = 0.002), glycemia response to glucose (r = 0.59; p = 0.001) and insulin (r = 0.42; p<0.05) injections (Fig 14A, 14B and 14C). Cell proliferation was negatively correlated with fasting glycemia (r = 0.39; p<0.05) (Fig 14D).

## Discussion

The results show that multiple infusions of allogeneic BM-MSCs are able to promote prolonged decrease in glucose intolerance and apoptosis in pancreatic islets and increase in insulin sensitivity in hyperglycemic HFD fed mice. To the best of our knowledge this is the first report of the effects of BM-MSCs infusion in pancreatic and metabolic parameters in exclusively HFD fed hyperglycemic mice.

The HFD fed rodent is one of the most widely used models for studying the metabolic derangements caused by obesity in humans, including glucose intolerance, insulin resistance and type 2 diabetes mellitus. It had been previously described that *Swiss* mice fed HFD for 8 weeks develop obesity, insulin resistance and hyperglycemia as observed in the present study [30]. This animal model resembles more closely the human type 2 diabetes background since it has no transgenic manipulations or drug-induced β cell toxicity, such as caused by streptozotocin.

Previous studies have reported beneficial effects of stem cells transplantation on the metabolic status in both types 1 and 2 diabetic patients and in rodent models of diabetes [5,13,14,15,16,17,20,22,31,32,33,34,35,36,37,38,39]. The beneficial effects have been attributed to immunological and regenerative properties of different origin stem cells, including umbilical cord cells [40,41,42,43]. However, most pre-clinical studies use streptozotocin-induced diabetes models to evaluate the metabolic effects of single or multiple intravenous MSCs infusions. To the best of our knowledge this is the first report of the effects of BM-MSCs infusion in pancreatic and metabolic parameters in exclusively HFD fed hyperglycemic mice.

Several studies suggest that MSCs may be autologously, allogeinic and even xenogeinic transplanted since the infusion of MSCs in immunocompetent animal models did not lead to rejection of the same and provided good therapeutic results [17,38,42,44,45].

In the present study, the progressive decrease of fasting glycemia in HFD-MSCs-R animals was observed since the 7^th^ week post-MSCs infusions and maintained until the 16^th^ week post-MSCs infusions. At this time, there was no difference in glucose levels between C animals and HFD-MSC-R animals. It was previously demonstrated that MSCs infusion in only STZ or STZ+HFD diabetes animal models is accompanied by decrease of hyperglycemia as observed in the present study, however in most of the studies, the experimental period was shorter than the one in the present study, even though in most of the studies, the experimental period was shorter than in the present study [13,14,15,16,17,20,22,38] and there was increase of plasma insulin levels[14,15,20,22,38].

In spite of the consistent decrease of hyperglycemia promoted by the multiple MSCs infusions, morphometric analysis of the islets showed that the total islet area and the relative volume of β-cell did not change post-MSCs infusions, as opposed to previous reports using other animal models [13,14,15,16,22]. This result associated with the unchanged insulin levels and the decreased glycemia response to both, glucose and insulin injections suggest the increase of insulin sensitivity of peripheral target tissues in the animals treated with MSCs, as recently reported by others [14,38]. Indeed, it was demonstrated that MSC infusion increased expression of GLUT4 and elevated phosphorilation of insulin receptor 1 and protein kinase B in insulin target tissues [14,39]. The mechanisms, however, are poorly understood. It is speculated that the migration of MSCs to different organs [46], in special to damaged tissues [47], could lead to modulation of insulin resistance and local inflammation, which are typical mechanisms involved in pathogenesis of obesity and type 2 diabetes. Most evidences suggest that the beneficial effects of BM-MSCs transplantation are not associated with trans-differentiation of MSCs into pancreatic β cells but rather related to MSC-derived paracrine factors such as cytokines and growth factorsresponsible for immunossupression, differentiation, angiogenesis and stimulation of endogenous cells [48,49,50]. The migration and homing ability of MSCs to injured tissues enable the pacrine effects of MSCs [51]. Even though we did not evaluate MSCs migration and homing analysis in the present study, previous studies have shown that MSCs injected intraperitoneally migrate to various organs such as lung, spleen, pancreas, kidneys, brain, heart, thymus, liver, among others [52,53].

The data regarding the pattern of α-cells and glucagon in pathologic states such as obesity, insulin resistance and type 2 diabetes in both humans and rodents are highly controversial [54,55,56,57,58,59,60]. In the present study, the volume of α-cells decreased in HFD fed mice. This finding could be associated with increased insulin levels since insulin is a potent α-cell inhibitor [59,60,61,62]. In addition, recent evidences point to the conversion of pancreatic α-cells to β-cells in conditions of stress [63,64]. A substantial proportion of α-cells becoming β-cells could explain the decrease in α-cells volume in the presence of unaltered β-cell observed in HFD. Nevertheless, this hypothesis does not explain the increase in α-cells volume observed in HFD-MSCs-NR group in comparison with HFD-MSCs-R group.

Beta-cell apoptosis has been considered an important mechanism in the pathogenesis of type 2 diabetes and increased islet apoptosis in HFD fed mice was demonstrated [65]. In the present study, not only did apoptosis increase in HFD fed mice but also proliferation decreased in these animals. Although islet proliferation did not change after MSCs infusions, islet apoptosis decreased in HFD-MSCs-R group. This result is confirmed by an *in vitro* study that demonstrates decreased caspase 3 expression and new expression of Ki67 in islet cell nuclei by electrofusion between dispersed islet cells and MSCs [66]. In addition, it was recently demonstrated *in vitro* that MSCs co-cultured with pancreatic islets release trophic factors that increase the survival of the islets and lead to expression of Pdx1 [67]. Altogether these data emphasize the cytoprotective property of MSCs [11]. Interestingly, islet apoptosis was positively correlated with fasting glycemia and glucose response to both glucose and insulin injections and islet proliferation was negatively correlated with fasting glycemia. These findings demonstrated the importance of the entire islet cell population and metabolic modulation.

In conclusion, the results demonstrate the ability of MSCs to promote prolonged decrease in hyperglycemia and apoptosis in pancreatic islets and increase in insulin sensitivity in HFD fed mice.

## References

[1] Stumvoll M, Goldstein BJ, van Haeften TW. Type 2 diabetes: principles of pathogenesis and therapy. Lancet. 2005; 365: 1333–1346. 15823385 10.1016/S0140-6736(05)61032-X

[2] Asghar Z, Yau D, Chan F, Leroith D, Chan CB, Wheeler MB. Insulin resistance causes increased beta-cell mass but defective glucose-stimulated insulin secretion in a murine model of type 2 diabetes. Diabetologia. 2006; 49: 90–99. 16362284 10.1007/s00125-005-0045-y

[3] Butler AE, Janson J, Soeller WC, Butler PC. Increased beta-cell apoptosis prevents adaptive increase in beta-cell mass in mouse model of type 2 diabetes: evidence for role of islet amyloid formation rather than direct action of amyloid. Diabetes. 2003; 52: 2304–2314. 12941770 10.2337/diabetes.52.9.2304

[4] Zenari L, Marangoni A. What are the preferred strategies for control of glycaemic variability in patients with type 2 diabetes mellitus? Diabetes Obes Metab. 2013; 15 Suppl 2: 17–25. 10.1111/dom.12143 24034516

[5] Hess D, Li L, Martin M, Sakano S, Hill D, Strutt B, et al Bone marrow-derived stem cells initiate pancreatic regeneration. Nat Biotechnol. 2003; 21: 763–770. 12819790 10.1038/nbt841

[6] Si YL, Zhao YL, Hao HJ, Fu XB, Han WD. MSCs: Biological characteristics, clinical applications and their outstanding concerns. Ageing Res Rev. 2011; 10: 93–103. 10.1016/j.arr.2010.08.005 20727988

[7] Aggarwal S, Pittenger MF. Human mesenchymal stem cells modulate allogeneic immune cell responses. Blood. 2005; 105: 1815–1822. 15494428 10.1182/blood-2004-04-1559

[8] Levesque JP, Winkler IG, Larsen SR, Rasko JE. Mobilization of bone marrow-derived progenitors. Handb Exp Pharmacol. 2007: 3–36. 17554502 10.1007/978-3-540-68976-8_1

[9] Nauta AJ, Fibbe WE. Immunomodulatory properties of mesenchymal stromal cells. Blood. 2007; 110: 3499–3506. 17664353 10.1182/blood-2007-02-069716

[10] Park KS, Kim YS, Kim JH, Choi B, Kim SH, Tan AH, et al Trophic molecules derived from human mesenchymal stem cells enhance survival, function, and angiogenesis of isolated islets after transplantation. Transplantation. 2010; 89: 509–517. 10.1097/TP.0b013e3181c7dc99 20125064

[11] Yeung TY, Seeberger KL, Kin T, Adesida A, Jomha N, Shapiro AM, et al Human mesenchymal stem cells protect human islets from pro-inflammatory cytokines. PLoS One. 2012; 7: e38189 10.1371/journal.pone.0038189 22666480 PMC3364233

[12] Stagg J. Immune regulation by mesenchymal stem cells: two sides to the coin. Tissue Antigens. 2006; 69: 1–9. 10.1111/j.1399-0039.2006.00739.x 17212702

[13] Ezquer FE, Ezquer ME, Parrau DB, Carpio D, Yanez AJ, Conget PA. Systemic administration of multipotent mesenchymal stromal cells reverts hyperglycemia and prevents nephropathy in type 1 diabetic mice. Biol Blood Marrow Transplant. 2008; 14: 631–640. 10.1016/j.bbmt.2008.01.006 18489988

[14] Si Y, Zhao Y, Hao H, Liu J, Guo Y, Mu Y, et al Infusion of mesenchymal stem cells ameliorates hyperglycemia in type 2 diabetic rats: identification of a novel role in improving insulin sensitivity. Diabetes. 2012; 61: 1616–1625. 10.2337/db11-1141 22618776 PMC3357293

[15] Ezquer F, Ezquer M, Simon V, Conget P. The antidiabetic effect of MSCs is not impaired by insulin prophylaxis and is not improved by a second dose of cells. PLoS One. 2011; 6: e16566 10.1371/journal.pone.0016566 21304603 PMC3029393

[16] Dinarvand P, Hashemi SM, Soleimani M. Effect of transplantation of mesenchymal stem cells induced into early hepatic cells in streptozotocin-induced diabetic mice. Biol Pharm Bull. 2010; 33: 1212–1217. 20606315 10.1248/bpb.33.1212

[17] Lee RH, Seo MJ, Reger RL, Spees JL, Pulin AA, Olson SD, et al Multipotent stromal cells from human marrow home to and promote repair of pancreatic islets and renal glomeruli in diabetic NOD/scid mice. Proc Natl Acad Sci U S A. 2006; 103: 17438–17443. 17088535 10.1073/pnas.0608249103 PMC1634835

[18] Lin P, Chen L, Yang N, Sun Y, Xu YX. Evaluation of stem cell differentiation in diabetic rats transplanted with bone marrow mesenchymal stem cells. Transplant Proc. 2009; 41: 1891–1893. 10.1016/j.transproceed.2009.02.078 19545751

[19] Zhou H, Tian HM, Long Y, Zhang XX, Zhong L, Deng L, et al Mesenchymal stem cells transplantation mildly ameliorates experimental diabetic nephropathy in rats. Chin Med J (Engl). 2009; 122: 2573–2579. 19951572

[20] Boumaza I, Srinivasan S, Witt WT, Feghali-Bostwick C, Dai Y, Garcia-Ocana A, et al Autologous bone marrow-derived rat mesenchymal stem cells promote PDX-1 and insulin expression in the islets, alter T cell cytokine pattern and preserve regulatory T cells in the periphery and induce sustained normoglycemia. *J Autoimmun*. 2009; 32: 33–42. 19062254 10.1016/j.jaut.2008.10.004

[21] Sordi V, Malosio ML, Marchesi F, Mercalli A, Melzi R, Giordano T, et al Bone marrow mesenchymal stem cells express a restricted set of functionally active chemokine receptors capable of promoting migration to pancreatic islets. Blood. 2005; 106: 419–427. 15784733 10.1182/blood-2004-09-3507

[22] Hao H, Liu J, Shen J, Zhao Y, Liu H, Hou Q, et al Multiple intravenous infusions of bone marrow mesenchymal stem cells reverse hyperglycemia in experimental type 2 diabetes rats. Biochem Biophys Res Commun. 2013; 436: 418–423. 10.1016/j.bbrc.2013.05.117 23770360

[23] Hu J, Wang F, Sun R, Wang Z, Yu X, Wang L, et al Effect of combined therapy of human Wharton's jelly-derived mesenchymal stem cells from umbilical cord with sitagliptin in type 2 diabetic rats. Endocrine. 2014; 45: 279–287. 10.1007/s12020-013-9984-0 23686639

[24] Hellman B. The total volume of the pancreatic islet tissue at different ages of the rat. Acta Pathol Microbiol Scand. 1959; 47: 35–50. 14400892 10.1111/j.1699-0463.1959.tb03419.x

[25] Butler AE, Janson J, Bonner-Weir S, Ritzel R, Rizza RA, Butler PC. Beta-cell deficit and increased beta-cell apoptosis in humans with type 2 diabetes. Diabetes. 2003; 52: 102–110. 12502499 10.2337/diabetes.52.1.102

[26] Urban VS, Kiss J, Kovacs J, Gocza E, Vas V, Monostori E, et al Mesenchymal stem cells cooperate with bone marrow cells in therapy of diabetes. Stem Cells. 2008; 26: 244–253. 17932424 10.1634/stemcells.2007-0267

[27] Laschke MW, Schank TE, Scheuer C, Kleer S, Shadmanov T, Eglin D, et al In vitro osteogenic differentiation of adipose-derived mesenchymal stem cell spheroids impairs their in vivo vascularization capacity inside implanted porous polyurethane scaffolds. Acta Biomater. 2014. 10.1016/j.actbio.2014.06.035 24998773

[28] Pittenger MF, Mackay AM, Beck SC, Jaiswal RK, Douglas R, Mosca JD, et al Multilineage potential of adult human mesenchymal stem cells. Science. 1999; 284: 143–147. 10102814 10.1126/science.284.5411.143

[29] Cheng CC, Lian WS, Hsiao FS, Liu IH, Lin SP, Lee YH, et al Isolation and characterization of novel murine epiphysis derived mesenchymal stem cells. PLoS One. 2012; 7: e36085 10.1371/journal.pone.0036085 22558340 PMC3338631

[30] De Souza CT, Araujo EP, Stoppiglia LF, Pauli JR, Ropelle E, Rocco SA, et al Inhibition of UCP2 expression reverses diet-induced diabetes mellitus by effects on both insulin secretion and action. FASEB J. 2007; 21: 1153–1163. 17209127 10.1096/fj.06-7148com

[31] Voltarelli JC, Couri CE, Stracieri AB, Oliveira MC, Moraes DA, Pieroni F, et al Autologous nonmyeloablative hematopoietic stem cell transplantation in newly diagnosed type 1 diabetes mellitus. JAMA. 2007; 297: 1568–1576. 17426276 10.1001/jama.297.14.1568

[32] Voltarelli JC, Couri CE. Stem cell transplantation for type 1 diabetes mellitus. Diabetol Metab Syndr. 2009; 1: 4 10.1186/1758-5996-1-4 19825196 PMC2758595

[33] Hasegawa Y, Ogihara T, Yamada T, Ishigaki Y, Imai J, Uno K, et al Bone marrow (BM) transplantation promotes beta-cell regeneration after acute injury through BM cell mobilization. Endocrinology. 2007; 148: 2006–2015. 17255204 10.1210/en.2006-1351

[34] Jiang R, Han Z, Zhuo G, Qu X, Li X, Wang X, et al Transplantation of placenta-derived mesenchymal stem cells in type 2 diabetes: a pilot study. Front Med. 2011; 5: 94–100. 10.1007/s11684-011-0116-z 21681681

[35] Bhansali A, Upreti V, Khandelwal N, Marwaha N, Gupta V, Sachdeva N, et al Efficacy of autologous bone marrow-derived stem cell transplantation in patients with type 2 diabetes mellitus. Stem Cells Dev. 2009; 18: 1407–1416. 10.1089/scd.2009.0164 19686048

[36] Ezquer F, Ezquer M, Contador D, Ricca M, Simon V, Conget P. The antidiabetic effect of mesenchymal stem cells is unrelated to their transdifferentiation potential but to their capability to restore TH1/TH2 balance and to modify the pancreatic microenvironment. Stem Cells. 2012; 30: 1664–1674. 10.1002/stem.1132 22644660

[37] Gao X, Song L, Shen K, Wang H, Niu W, Qin X. Transplantation of bone marrow derived cells promotes pancreatic islet repair in diabetic mice. Biochem Biophys Res Commun. 2008; 371: 132–137. 10.1016/j.bbrc.2008.04.033 18420028

[38] Ho JH, Tseng TC, Ma WH, Ong WK, Chen YF, Chen MH, et al Multiple intravenous transplantations of mesenchymal stem cells effectively restore long-term blood glucose homeostasis by hepatic engraftment and beta-cell differentiation in streptozocin-induced diabetic mice. Cell Transplant. 2012; 21: 997–1009. 10.3727/096368911X603611 22004871

[39] Hughey CC, Ma L, James FD, Bracy DP, Wang Z, Wasserman DH, et al Mesenchymal stem cell transplantation for the infarcted heart: therapeutic potential for insulin resistance beyond the heart. Cardiovasc Diabetol. 2013; 12: 128 10.1186/1475-2840-12-128 24007410 PMC3847505

[40] Francese R, Fiorina P. Immunological and regenerative properties of cord blood stem cells. Clin Immunol. 2010; 136: 309–322. 10.1016/j.clim.2010.04.010 20447870

[41] Jurewicz M, Yang S, Augello A, Godwin JG, Moore RF, Azzi J, et al Congenic mesenchymal stem cell therapy reverses hyperglycemia in experimental type 1 diabetes. Diabetes. 2010; 59: 3139–3147. 10.2337/db10-0542 20841611 PMC2992776

[42] Fiorina P, Jurewicz M, Augello A, Vergani A, Dada S, La Rosa S, et al Immunomodulatory function of bone marrow-derived mesenchymal stem cells in experimental autoimmune type 1 diabetes. J Immunol. 2009; 183: 993–1004. 10.4049/jimmunol.0900803 19561093 PMC3895445

[43] Fiorina P, Voltarelli J, Zavazava N. Immunological applications of stem cells in type 1 diabetes. Endocr Rev. 2011; 32: 725–754. 10.1210/er.2011-0008 21862682 PMC3591677

[44] Zhang J, Li Y, Chen J, Cui Y, Lu M, Elias SB, et al Human bone marrow stromal cell treatment improves neurological functional recovery in EAE mice. Exp Neurol. 2005; 195: 16–26. 15904921 10.1016/j.expneurol.2005.03.018

[45] Kerkis I, Ambrosio CE, Kerkis A, Martins DS, Zucconi E, Fonseca SA, et al Early transplantation of human immature dental pulp stem cells from baby teeth to golden retriever muscular dystrophy (GRMD) dogs: Local or systemic? J Transl Med. 2008; 6: 35 10.1186/1479-5876-6-35 18598348 PMC2529267

[46] Gao J, Dennis JE, Muzic RF, Lundberg M, Caplan AI. The dynamic in vivo distribution of bone marrow-derived mesenchymal stem cells after infusion. Cells Tissues Organs. 2001; 169: 12–20. 11340257 10.1159/000047856

[47] Le Blanc K, Pittenger M. Mesenchymal stem cells: progress toward promise. Cytotherapy. 2005; 7: 36–45. 16040382 10.1080/14653240510018118

[48] Xu YX, Chen L, Wang R, Hou WK, Lin P, Sun L, et al Mesenchymal stem cell therapy for diabetes through paracrine mechanisms. Med Hypotheses. 2008; 71: 390–393. 10.1016/j.mehy.2008.03.046 18538944

[49] Choi JB, Uchino H, Azuma K, Iwashita N, Tanaka Y, Mochizuki H, et al Little evidence of transdifferentiation of bone marrow-derived cells into pancreatic beta cells. Diabetologia. 2003; 46: 1366–1374. 12898006 10.1007/s00125-003-1182-9

[50] Burdon TJ, Paul A, Noiseux N, Prakash S, Shum-Tim D. Bone marrow stem cell derived paracrine factors for regenerative medicine: current perspectives and therapeutic potential. Bone Marrow Res. 2011; 2011: 207326 10.1155/2011/207326 22046556 PMC3195349

[51] Sohni A, Verfaillie CM. Mesenchymal stem cells migration homing and tracking. Stem Cells Int. 2013; 2013: 130763 10.1155/2013/130763 24194766 PMC3806396

[52] Meyerrose TE, De Ugarte DA, Hofling AA, Herrbrich PE, Cordonnier TD, Shultz LD, et al In vivo distribution of human adipose-derived mesenchymal stem cells in novel xenotransplantation models. Stem Cells. 2007; 25: 220–227. 16960135 10.1634/stemcells.2006-0243 PMC4382309

[53] Wilson T, Stark C, Holmbom J, Rosling A, Kuusilehto A, Tirri T, et al Fate of bone marrow-derived stromal cells after intraperitoneal infusion or implantation into femoral bone defects in the host animal. J Tissue Eng. 2010; 2010: 345806 10.4061/2010/345806 21350643 PMC3042670

[54] Li Z, Karlsson FA, Sandler S. Islet loss and alpha cell expansion in type 1 diabetes induced by multiple low-dose streptozotocin administration in mice. J Endocrinol. 2000; 165: 93–99. 10750039 10.1677/joe.0.1650093

[55] Fraulob JC, Ogg-Diamantino R, Fernandes-Santos C, Aguila MB, Mandarim-de-Lacerda CA. A Mouse Model of Metabolic Syndrome: Insulin Resistance, Fatty Liver and Non-Alcoholic Fatty Pancreas Disease (NAFPD) in C57BL/6 Mice Fed a High Fat Diet. J Clin Biochem Nutr. 2010; 46: 212–223. 10.3164/jcbn.09-83 20490316 PMC2872226

[56] Henquin JC, Rahier J. Pancreatic alpha cell mass in European subjects with type 2 diabetes. Diabetologia. 2011; 54: 1720–1725. 10.1007/s00125-011-2118-4 21465328 PMC3110273

[57] Meier JJ, Ueberberg S, Korbas S, Schneider S. Diminished glucagon suppression after beta-cell reduction is due to impaired alpha-cell function rather than an expansion of alpha-cell mass. Am J Physiol Endocrinol Metab. 2011; 300: E717–723. 10.1152/ajpendo.00315.2010 21285404 PMC3279300

[58] Schwasinger-Schmidt T, Robbins DC, Williams SJ, Novikova L, Stehno-Bittel L. Long-term liraglutide treatment is associated with increased insulin content and secretion in beta-cells, and a loss of alpha-cells in ZDF rats. Pharmacol Res. 2013; 76: 58–66. 10.1016/j.phrs.2013.07.005 23891763

[59] Kilimnik G, Zhao B, Jo J, Periwal V, Witkowski P, Misawa R, et al Altered islet composition and disproportionate loss of large islets in patients with type 2 diabetes. PLoS One. 2011; 6: e27445 10.1371/journal.pone.0027445 22102895 PMC3216964

[60] Gosmain Y, Masson MH, Philippe J. Glucagon: the renewal of an old hormone in the pathophysiology of diabetes. J Diabetes. 2013; 5: 102–109. 10.1111/1753-0407.12022 23302052

[61] Quesada I, Tuduri E, Ripoll C, Nadal A. Physiology of the pancreatic alpha-cell and glucagon secretion: role in glucose homeostasis and diabetes. J Endocrinol. 2008; 199: 5–19. 10.1677/JOE-08-0290 18669612

[62] Habener JF, Stanojevic V. alpha-cell role in beta-cell generation and regeneration. Islets. 2012; 4: 188–198. 10.4161/isl.20500 22847495 PMC3442816

[63] Thorel F, Herrera PL. [Conversion of adult pancreatic alpha-cells to beta-cells in diabetic mice]. Med Sci (Paris). 2010; 26: 906–909. 10.1051/medsci/20102611906 21106168

[64] Chung CH, Levine F. Adult pancreatic alpha-cells: a new source of cells for beta-cell regeneration. Rev Diabet Stud. 2010; 7: 124–131. 10.1900/RDS.2010.7.124 21060971 PMC2989785

[65] Sone H, Kagawa Y. Pancreatic beta cell senescence contributes to the pathogenesis of type 2 diabetes in high-fat diet-induced diabetic mice. Diabetologia. 2005; 48: 58–67. 15624098 10.1007/s00125-004-1605-2

[66] Yanai G, Hayashi T, Zhi Q, Yang KC, Shirouzu Y, Shimabukuro T, et al Electrofusion of mesenchymal stem cells and islet cells for diabetes therapy: a rat model. PLoS One. 2013; 8: e64499 10.1371/journal.pone.0064499 23724055 PMC3665804

[67] Scuteri A, Donzelli E, Rodriguez-Menendez V, Ravasi M, Monfrini M, Bonandrini B, et al A double mechanism for the mesenchymal stem cells' positive effect on pancreatic islets. PLoS One. 2014; 9: e84309 10.1371/journal.pone.0084309 24416216 PMC3885554

